# Effect of CYP2D6 polymorphisms on plasma concentration and therapeutic effect of risperidone

**DOI:** 10.1186/s12888-020-03034-9

**Published:** 2021-02-03

**Authors:** Jinjun Lu, Ye Yang, Jian Lu, Zuqing Wang, Yiping He, Yeliang Yan, Kai Fu, Wenjuan Jiang, Yunqing Xu, Renrong Wu, Wenqing Liu, Jingping Zhao

**Affiliations:** 1 The Third People’s Hospital of Jiangyin City, Wuxi, Jiangsu Province China; 2 National Clinical Research Center for Mental Disorders, and Department of Psychiatry, The Second Xiangya Hospital of Central South University, Changsha, 410011 Hunan China

**Keywords:** *CYP2D6*, Polymorphisms, Risperidone, Plasma concentration, Therapeutic effect

## Abstract

**Background:**

This study aimed to investigate the influence of *CYP2D6* polymorphisms on risperidone plasma concentrations in patients with schizophrenia. Based on pharmacogenomics, we examined whether plasma concentration of risperidone is associated with clinical response and adverse side-effects.

**Methods:**

We recruited patients with chronic schizophrenia who were then treated with risperidone. The *CYP2D6* genotypes were determined using targeted sequencing. All high-frequency mutation sites of the nine exons of the gene were assayed in the present study. Plasma concentrations of risperidone and 9-hydroxyrisperidone (9-OH-RIS) were measured using high-performance liquid chromatography (HPLC). Psychiatric symptoms were monitored using The Positive and Negative Syndrome Scale (PANSS), Brief Psychiatric Rating Scale (BPRS), and Clinical Global Impression (CGI). Adverse effects were evaluated using the Barnes Akathisia Scale (BAS) and Extrapyramidal Symptom Rating Scale (ESRS). Follow-up visits were scheduled at weeks 2,4, and 8 after treatment initiation.

**Results:**

Among the 76 patients, 100 C > T (rs1065852), 1038 C > T (rs1081003), 1662 G > C (rs1058164), 2851 C > T (rs16947), and 4181G > C (rs1135840) variants were detected. The most common allele was CYP2D6*10 (81.6%), whereas CYP2D6*2 (9.2%) and CYP2D6*5 (17.1%) were relatively rare. Plasma levels of risperidone and the risperidone/9-OH risperidone ratio (R/9-OH) were significantly increased in individuals with CYP2D6*10 (*P* < 0.05). The change in PANSS score, weight, high-density lipoprotein (HDL) level, prolactin (PRL) level, and ESRS were significantly different from baseline, between the different genotypes (*P* < 0.01). Moreover, individuals with CYP2D6*10 homozygous (TT) mutations were associated with higher risperidone concentration and R/9-OH ratio than those with heterozygous mutations (CT) (*P* < 0.01). A change from baseline in BPRS scores was observed only during week 8 and was different between heterozygous and homozygous mutations. As for the C2851T polymorphism, the incidence of adverse metabolic effects was significantly different between the C/C and C/T genotypes (*P* < 0.01). Regarding the *G4181C* polymorphisms, the changes from baseline in GLU and TG, were different between the C/C and C/G genotypes (*P* < 0.01).

**Conclusions:**

The genotype of *CYP2D6* significantly influences the plasma concentration of risperidone and may subsequently influence the adverse side-effects following risperidone treatment, while also exerting a slight influence on clinical outcomes.

**Supplementary Information:**

The online version contains supplementary material available at 10.1186/s12888-020-03034-9.

## Background

Risperidone, an atypical antipsychotic, is widely used for the treatment of psychiatric problems and is associated with better compliance and reduced incidence of adverse reactions compared with typical antipsychotics [1]. Although risperidone has shown to be more effective relative to other antipsychotics, there are still some patients with psychiatric problems who do not respond to any antipsychotic therapeutics [2, 3]. Such situations can lead to patients having persistent exposure to antipsychotic drugs, which can be accompanied by serious side-effects, increased discomfort, and ultimately resulting in poor treatment adherence [4, 5]. Among different patients, considerable clinical heterogeneity has been observed in the effects of risperidone, which affects the efficacy of treatment for mental illness [6]. Understanding the range of treatment responses and limiting risperidone exposure to within the therapeutic range, are important factors for achieving optimal treatment outcomes.

Risperidone is predominantly metabolized by the cytochrome P450 enzymes *CYP2D6* and *CYP3A4* [7]. The gene encoding *CYP2D6* exhibits high polymorphism, and allelic variants are considered to influence the metabolic pathway of antipsychotics [8]. The main active metabolite of risperidone is 9-hydroxyrisperidone (9-OH-RIS), which exhibits similar pharmacological actions to risperidone [9]. Therefore, it is suggested that the therapeutic response of risperidone can be influenced by the actions of plasma risperidone and the concentration of 9-OH-RIS [6]. Indeed, there is emerging evidence highlighting the heterogeneity of plasma concentrations of risperidone and 9-OH-RIS, as well as treatment response, between patients with different *CYP2D6 g*enotypes [10, 11]. Significantly higher risperidone levels and risperidone/9-OH-RIS(R/9-OH) ratios have been reported in patients carrying the CYP2D6*10/*10 variant compared with other alleles [12]. Similar results have been reported for children with autism spectrum disorders treated with risperidone, in that those with the CYP2D6*5/*10, CYP2D6*10/*10, or CYP2D6*10/*41 alleles exhibited higher levels of risperidone after administration [13]. These observations indicate that specific *CYP2D6* variants influence the pharmacokinetics of risperidone. Further, an apparent association has been reported between *CYP2D6* polymorphisms and clinical improvement in response to risperidone. Specifically, significant clinical improvements, assessed via the Positive and Negative Syndrome Scale – Thai (PANSS-T), have been reported in patients with the *CYP2D6* “poor-metabolizer” phenotype compared with treatment outcomes in “extensive” metabolizers [14]. However, this result has not been verified and the conclusions are still largely controversial. A previous study involving 136 patients with psychotic disorders treated with a single-dose of risperidone, did not identify any significant association between *CYP2D6* polymorphisms and clinical recovery [15], which was supported by another study involving female patients with schizophrenia that found that PANSS improved following risperidone treatment, but was not associated with *CYP2D6* genotype [16]. These studies highlight the discrepancies in the literature relating to *CYP2D6*, which may originate from the different genotypes that were analyzed in different studies. To the best of our knowledge, there have been no comprehensive studies exploring the effects of *CYP2D6* variations on clinical outcomes of risperidone treatment.

Adverse effects, in particular metabolic-related effects, can contribute to reduced patient compliance irrespective of symptomatic improvement [17]. The risk of adverse metabolic effects in response to risperidone has been shown to vary greatly between individuals [11], indicating that genetics might play a significant role in susceptibility to adverse metabolic effects, indeed, CYP2D6*10 has been reported to be significantly associated with weight gain after risperidone treatment [17]. Other adverse side-effects, such as elevated prolactin and extrapyramidal syndrome (EPS), have also been reported in association with particular *CYP2D6* variants [18, 19]. However, in adult populations, the association between *CYP2D6* polymorphisms and serum prolactin concentrations are unclear and whether this association exists in patients with schizophrenia, remains to be investigated. Discrepancies have also been reported regarding the relationship between *CYP2D6* polymorphisms and EPS, with several studies failing to detect any significant difference in the incidence of EPS in association with *CYP2D6* variations, while some studies reported only a marginally significant trend [20, 21]. The effects of *CYP2D6* polymorphism on EPS in response to risperidone are unclear and require further exploration.

It is clear that genotypic differences contribute to the discrepancies observed in clinical outcomes. We detected all mutations in nine exons of the full-length *CYP2D6* sequence, which was further investigated in order to obtain a more comprehensive understanding of *CYP2D6* polymorphisms. The present study aimed to investigate whether *CYP2D6* polymorphisms significantly effect serum/plasma concentrations of risperidone in a clinical setting. To this end, we used pharmacogenomics to investigate whether plasma concentrations of risperidone are associated with clinical responses and the incidence of adverse effects, including adverse metabolic effects and EPS.

## Methods

### Patients and methods

We recruited patients that were all between 18 to 68 years of age and had been diagnosed with chronic schizophrenia (according to the criteria of the International Classification of Diseases-Tenth Edition [ICD-10]), within the last 5 years, at Third People’s Hospital of Jiangyin City, Jiangsu Province between May 2018, and May 2019. All patients had to meet the following criteria: 1) had not taken any antipsychotics drugs for at least 1 year; 2) had an acute attack and were re-admitted to the hospital; and 3) were treated with risperidone monotherapy after hospitalization. Risperidone dosage was increased gradually from a low dose to a therapeutic dose within 1 week (3–6 mg/day). Exclusion criteria were as follows: 1) Had undergone electroconvulsive therapy within 3 months of study enrollment, 2) administration of other psychotropic medications, including antidepressants and mood stabilizers, 3) had obvious brain damage or serious physical illness, and 4) presented with any other condition that rendered the patient unsuitable for the trial (e.g., allergies to related drugs, enrollment in clinical trials for other drugs or devices within 3 months of starting of the study). In total, 79 patients were recruited in the current study; however, 3 patients were dropped out of the study owing to drug adjustment or non-compliance. Ultimately, 76 patients were enrolled for subsequent data analyses.

We collected data at the time of enrollment. Baseline data included demographic information, a comprehensive medical history, clinical evaluation of psychiatric symptoms, a physical examination (including weight and height), laboratory tests (including fasting low-density lipoprotein [LDL-C], triglyceride, cholesterol, high-density lipoprotein [HDL-C], glucose levels, prolactin levels, liver and renal function tests, and electrocardiography), and analysis of *CYP2D6* genotype. The PANSS, Brief Psychiatric Rating Scale (BPRS), and Clinical Global Impression (CGI) were employed to monitor psychiatric symptoms. Treatment outcome was measured by assessing the change in clinical-symptom-scale scores over the study period. The improvement of clinical symptoms was expressed by the changes on the PANSS, BPRS, CGI scale scores from the initial assessment (week 0) to week 4 and then at week 8. The Barnes Akathisia Scale (BAS) and Extrapyramidal Symptom Rating Scale (ESRS) were used to evaluate adverse effects. Follow-up visits were made at 2,4 and 8 weeks after the initiation of treatment. During each follow-up visit, all evaluations (the physical examination, laboratory tests, weight measurements, clinical symptoms, and assessments of adverse effects) were repeated. Additionally, the concentrations of risperidone and 9-OH-RIS were measured at each follow-up visit.

### Evaluation of plasma concentration of risperidone

Fasting blood samples were collected from patients at each follow-up visit. Blood draws were consistently performed between 7 and 8 am, to ensure consistency across patients. Plasma was stored at − 20 °C until analysis. Plasma concentrations of risperidone and 9-OH-RIS were determined using high-performance liquid chromatography (HPLC) with the Agilent 1260 High Performance Liquid Chromatograph (Agilent 1260 HPLC, USA). The ratio of risperidone to 9-OH-RIS (R/9-OH ratio) was calculated from the concentration of risperidone divided by the concentration of 9-OH-RIS (ng/mL) to reflect the activity of *CYP2D6*. The concentration/dose (C/D) ratio was calculated by dividing the total concentration of risperidone plus 9-OH-RIS (ng/mL) by risperidone dose (mg/day), to provide an index of drug elimination capacity.

### Genotyping

An additional 3–4 mL volume of blood was drawn once for pharmacogenetic analyses at baseline. The whole blood sample was stored at − 70 °C for subsequent extraction of genomic DNA. *CYP2D6* genotypes were determined in all the patients using PCR assays followed by targeted sequencing. *CYP2D6* has nine exons that were all assayed in the current study. The whole experimental process consisted of primer design, polymerase chain reaction (PCR), and sequencing. Four sets of upstream and downstream primers were designed with Oligo6 software to amplify the nine exons using genomic DNA as templates. Extraction of DNA was carried out using the YC-B nucleic acid extraction reagent (Hygeianecy Biological Company, Wuhan, China) according to manufacturer’s instructions. The DNA was extracted quickly then adsorbed onto a high-performance solid-phase matrix to obtain high-purity DNA by elution. The quality of genomic DNA underwent strict quality control including Nanodrop quantification and 1% agarose gel electrophoresis. The details of individual PCR programs were summarized in Supplemental Table 1. Finally, the 3730xl DNA Analyzer was used for sequencing (Applied Biosystems, USA). Only the five high-frequency mutation sites of the nine exons were detected, including 100C > T (rs1065852), 1038C > T (rs1081003), 1662G > C (rs1058164), 2851C > T mutations (rs16947), and 4181G > C mutations (rs1135840). The frequencies of mutations at the different sites are detailed in Table 1. The different mutation sites included 100C > T:62 (33TT,29CT), 1038C > T:62 (24TT,38CT), 1662C > G:68 (36CC,32CG), 2851C > T:20 (1TT,19CT), and 4181G > C:71 (43CC,28GC). These sites were categorized into CYP2D6*10, CYP2D6*2, and CYP2D6*65 according to the genotyping criteria for *CYP2D6* (Pharmacogene Variation Consortium, https://www.pharmvar.org/gene/*CYP2D6*).

**Table 1:** Demographic characteristics and genotypes of the study population

	*Total study population (n = 76)*
Sex (male/female)	38/38
Age (years)	45.0 (33.0, 54.0)
Height (cm)	164.5 (158.0, 171.0)
Weight (kg)	65.5 (56.3, 74.5)
BMI	23.3 (21.4, 27.9)
Dose (mg)	5.0 (4.0, 6.0)
PANSS	88.0 (82.0, 95.0)
BPRS	45.5 (41.3, 50.8)
CGI-S (2 week)	5.0 (5.0, 6.0)
CGS-I (2 week)	3.0 (3.0, 4.0)
Mutation sites	
100 (C > T)	62 (81.6)
1038 (C > T)	62 (81.6)
1662 (G > C)	68 (89.5)
2851 (C > T)	20 (26.3)
4181 (G > C)	71 (93.4)
Genotypes	
CYP2D6*10	47 (81.6)
CYP2D6*2	7 (9.2)
CYP2D6*65	13 (17.1)

Data are presented as median (interquartile range) or number (%)

*Abbreviation*: *BMI* Body mass index, *PANSS* Positive and Negative Syndrome Scale, *BPRS* Brief Psychiatric Rating Scale, *CGI-S* Clinical Global Impression-Severity, *CGI-I* Clinical Global Impression-Improvement

### Statistical analysis

Statistical analyses were carried out using the Statistical Package for Social Sciences (SPSS version 23, Chicago, IL, USA). Two-tailed *p*-values of < 0.05 were considered to be of statistical significance. Continuous data with normal distribution are presented as mean and standard deviation, while non-normally distributed data are presented as median and interquartile range. Analysis of variance was used to compare data with normal distribution, while data with a non-normal distribution were analyzed using a 2-tailed Mann-Whitney U test to compare between two groups, or the Kruskal-Wallis test to compare multiple groups. The chi-square test was used to test differences in the distribution of categorical variables. Further comparisons between groups were carried out using the Dunn-Bonferroni post-hoc test. Multiple linear regression analysis was performed to analyze the relationship between *CYP2D6* genotype and clinical response.

## Results

### Demographic and genotypic characteristics

The mean course of disease for chronic patients was approximately 13.48 (± 7.80) years. The study sample was comprised of 76 patients. Demographic and clinical data of the study population are summarized in Table 1. The risperidone dose ranged from 2 to 6 mg among the study population. All subjects completed the clinical symptom evaluations including PANSS, BPRS and CGI at baseline. All results are presented in Table 1.

The majority of patients carried the CYP2D6*10 allele. The 1038C > T (rs1081003) and 1662 G > C (rs1058164) mutations were nonsense mutations; therefore, they were excluded from subsequent analysis of single nucleotide polymorphisms. Given that the CYP2D6*10 allele is the most common allelic mutation, the heterozygous and homozygous mutations were further analyzed in our study.

### Relationships between genotype and drug concentration, clinical outcome, and adverse effects

#### Plasma concentration of risperidone in different genotypes

**Fig. 1:**
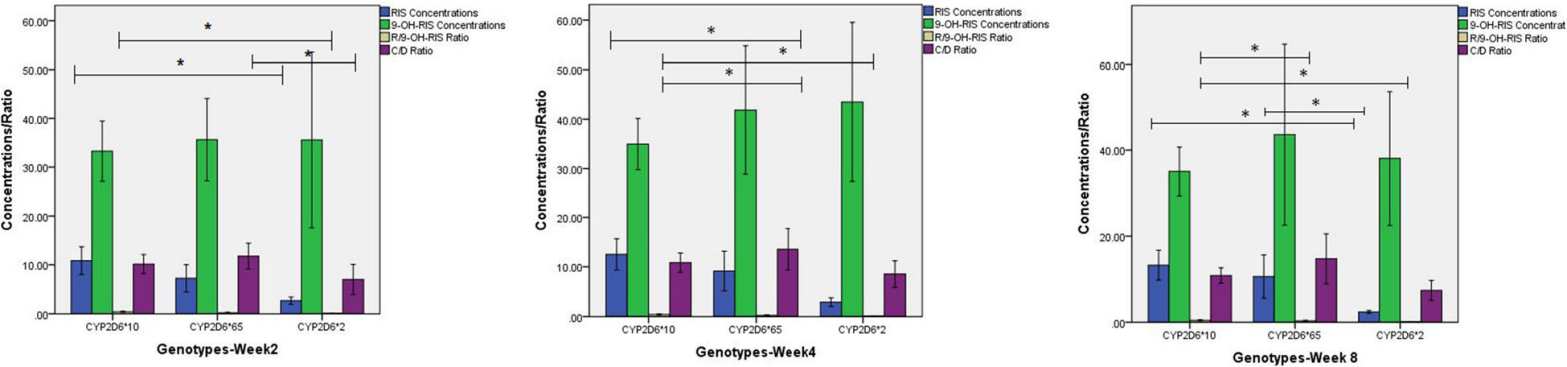
Bar graphs showing plasma concentrations of risperidone in relation to genotype. Abbreviations: C/D, concentration/dose ratio; RIS, risperidone; 9-OH-RIS, 9-hydroxyrisperidone; R/9-OH-RIS Ratio: risperidone/9-hydroxyrisperidone ratio; *p < 0.05

Figure 1 illustrates the plasma concentrations of risperidone, at each follow-up time point, in relation to genotype. Plasma/genotype interactions were significantly different between genotypes at each time point (all *P* < 0.05). The Kruskal-Wallis Test revealed an apparent association between *CYP2D6* genotype and plasma concentration of risperidone (all *P* < 0.05). Further pairwise comparisons revealed that, at all time points, plasma concentration of risperidone was significantly higher in subjects with the CYP2D6*10 genotype compared with those with the CYP2D6*2 genotype*,* after Bonferroni correction (all *P* < 0.05). Moreover, the plasma concentrations of risperidone in patients with the CYP2D6*65 and CYP2D6*2 genotypes were significantly different at week 8 (*Z* = 2.627, *P* = 0.026), while no significant differences were observed between subjects with the CYP2D6*65 and CYP2D6*10 alleles (all *P* > 0.05). We did not observe any difference in 9-OH-RIS between genotypes (all *P* > 0.05). The R/9-OH ratio was significantly higher in subjects with the CYP2D6*10 allele than those with the CYP2D6*2 allele, at all time points, and those with the CYP2D6*65 allele at 4 and 8 weeks (all *P* < 0.05 after correction). The C/D ratio at 2 weeks was significantly different between subjects with the *CYP2D6* genotype (*Z* = 7.126, *P* = 0.028), with a significant difference observed between CYP2D6*65 and CYP2D6*2 genotypes (*Z =* 2.642*, P* = 0.025); however, the strength of this correlation decreased over time with no significant association observed at 4 or 8 weeks (both *P* > 0.05).

### Relationship between polymorphisms of CYP2D6 and response to treatment

Symptom improvement in relation to genotype is presented in Table 2. The change of PANSS score from 0 to 8 weeks was significantly different between *CYP2D6* genotypes (*F =* 3.850*, P* = 0.027). PANSS scores were significantly higher in individuals with the CYP2D6*2 allele than in those with the CYP2D6*65 allele after pairwise comparison using Bonferroni’s post-hoc analysis (*P* = 0.007). CYP2D6*10 was also higher than CYP2D6*65; however, the difference was not statistically significant (*P* = 0.052). Additionally, no significant difference was observed between CYP2D6*10 and CYP2D6*2 (*P* = 0.112)*.* The BPRS and CGI scores were not significantly different among different genotypes from baseline to any follow-up visit (all *P* > 0.05).

**Table 2:** Relationships between *CYP2D6* genotype and clinical outcomes

	*Total study population (n = 76)*				
	CYP2D6*10	CYP2D6*2	CYP2D6*65	*Statistics*	*P*
PANSS0 - week 4	19.0 (12.0, 29.0)	24.0 (13.0, 21.0)	10.0 (7.5, 20.8)	5.328	0.070
PANSS0 - week 8	27.5 (19.0, 33.5)	34.5 (31.0, 39.5)	17.0 (13.0, 27.0)	3.850	0.027
BPRS0 – week 4	12.0 (8.0, 15.0)	12.0 (11.0, 13.0)	11.5 (6.3, 13.0)	1.059	0.589
BPRS0 - week 8	16.0 (13.0, 18.5)	15.0 (14.0, 18.0)	14.0(7.0, 17.0)	1.959	0.376
CGI-S0 - week 4	1.0 (1.0, 2.0)	2.0 (1.0, 2.0)	1.0 (1.0, 1.0)	2.853	0.240
CGI-S0 – week 8	2.0 (2.0, 3.0)	3.0 (2.0, 4.0)	2.0 (2.0, 3.0)	1.701	0.427
CGI-I2 – week 4	0.0 (0.0, 1.0)	0.0 (0.0, 0.0)	1.0 (0.0, 1.0)	2.523	0.283
CGI-I2 – week 8	1.0 (0.0, 1.0)	1.0 (0.0, 2.0)	1.0 (0.0, 1.5)	1.249	0.536

Data are presented as median (interquartile range)

PANSS0 - week 4 represents the change of PANSS score from 0 to 4 weeks

*Abbreviations*: *BPRS* Brief Psychiatric Rating Scale, *CGI* Clinical Global Impression, *CGI-S* Clinical Global Impression Severity, *CGI-I* Clinical Global Impression Improvement, *PANSS* Positive and Negative Syndrome Scale

#### Relationship between CYP2D6 polymorphisms and adverse effects

The relationships between *CYP2D6* polymorphisms and adverse effects are shown in Table 3. The change in weight from baseline to week 4 was significantly different between genotypes (*F =* 7.514*, P* = 0.001). Post-hoc tests for pairwise comparisons between the various genotypes identified significant differences between subjects with the CYP2D6*10 and CYP2D6*65 alleles (*P* < 0.001), and between those with the CYP2D6*65 and CYP2D6*2 (*P* = 0.025) alleles. The differences between HDL from baseline to week 8 were also significantly different between genotypes (*F =* 3.366*, P* = 0.042), and persisted between individuals with the CYP2D6*10 and CYP2D6*65 alleles, when between-group comparisons were conducted (*P* = 0.012). Prolactin levels were significantly associated with genotype (*F =* 4.359, *P* = 0.017). Post-hoc pairwise comparisons indicated that the change in prolactin levels in subjects with the CYP2D6*65 allele was significantly higher than other genotypes from baseline to week 8, especially the CYP2D6*10 allele (both *P* < 0.05). The occurrence of EPS was significantly different between different groups (*F =* 5.858, *p* = 0.037), which is mainly attributed to the higher occurrence of EPS in individuals with the CYP2D6*10 allele compared with the other two genotypes.

**Table 3:** Relationships between genotype and adverse effects

	CYP2D6*10	CYP2D6*2	CYP2D6*65	*Statistics*	*P*
Weight change from week 0 to 4, kg	−1.2 ± 3.0	−2.6 ± 2.5	−13.8 ± 23.4	7.514	0.001
Weight change from week 0 to 8, kg	−1.6 ± 3.6	−3.4 ± 2.0	−1.3 ± 3.8	0.798	0.455
BMI change from week 0 to 8	−0.6 ± 1.3	− 1.3 ± 0.8	− 0.5 ± 1.3	0.985	0.380
GLU change from week 0 to 4, mmol/l	− 0.5 ± 1.8	− 0.3 ± 0.4	−0.2 ± 2.8	0.102	0.903
GLU change from week 0 to 8, mmol/l	−0.3 ± 0.8	−0.6 ± 0.3	0.0 ± 1.4	0.596	0.554
TG change from week 0 to 4, mmol/l	0.0 ± 1.0	0.4 ± 1.1	−0.2 ± 0.6	0.994	0.376
TG change from week 0 to 8, mmol/l	−0.1 ± 1.0	−0.7 ± 1.6	−0.3 ± 0.7	1.800	0.175
CHO change from week 0 to 4, mmol/l	−0.3 ± 0.7	−0.1 ± 0.8	−0.6 ± 1.1	0.984	0.379
CHO change from week 0 to 8, mmol/l	−0.2 ± 0.8	−0.2 ± 0.8	−1.0 ± 2.0	2.395	0.100
LDL change from week 0 to 4, mmol/l	− 0.1 ± 0.5	− 0.1 ± 0.8	− 0.2 ± 0.5	0.964	0.387
LDL change from week0 to 8, mmol/l	− 0.1 ± 0.7	−0.1 ± 0.2	−0.5 ± 0.7	2.631	0.081
HDL change from week 0 to 4, mmol/l	−0.1 ± 0.2	−0.1 ± 0.2	−0.2 ± 0.2	1.041	0.359
HDL change from week 0 to 8, mmol/l	0.0 ± 0.2	−0.1 ± 0.2	−0.3 ± 0.5	3.366	0.042
PRL change from week 0 to 4, mmol/l	52.1 ± 62.9	63.4 ± 63.3	72.5 ± 79.8	0.509	0.603
PRL change from week 0 to 8, mmol/l	39.4 ± 53.7	80.0 ± 65.8	95.4 ± 76.1	4.359	0.017
ESRS week 4	8	0	5	5.858	0.037
ESRS week 8	11	1	4	0.930	0.633
BAS week 4	5	1	2	0.897	0.712
BAS week 8	6	1	1	0.493	0.632

Data are presented as mean ± standard deviation. *Abbreviations*: *BAS* Barnes Akathisia Scale, *BMI* Body mass index, *CHO* Cholesterol, *ESRS* Extrapyramidal Symptom Rating Scale, *GLU* Glucose, *HDL* High-density lipoprotein, *LDL* Low-density lipoprotein, *PRL* Prolactin, *TG* Triglyceride

### Differences in plasma concentration of risperidone and response to treatment between subjects with heterozygous or homozygous mutations of CYP2D6*10

**Fig. 2:**
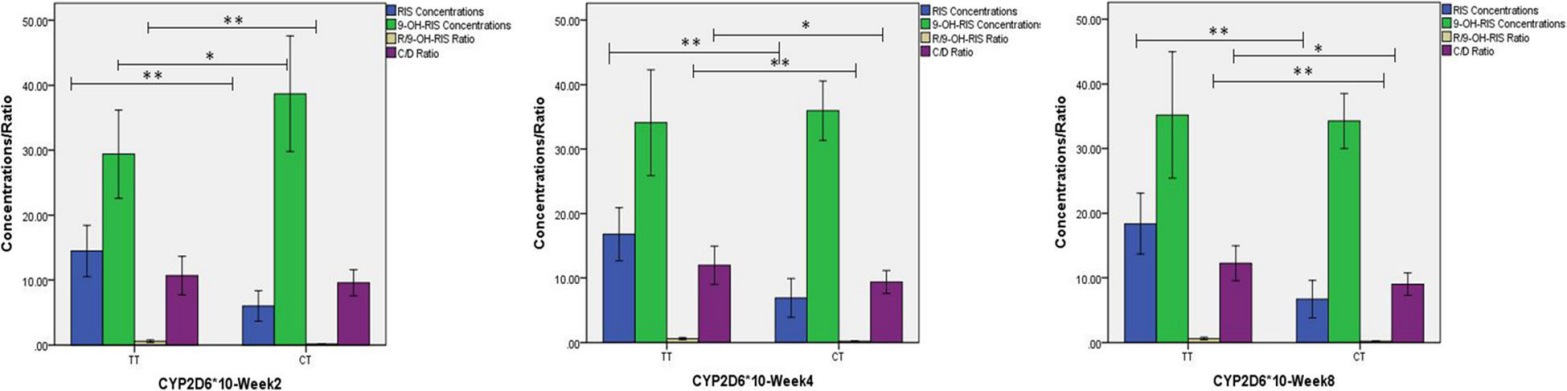
Bar graphs showing plasma concentrations of risperidone in subjects with heterozygous and homozygous mutations of CYP2D6*10. Abbreviations: C/D, concentration/dose ratio; RIS, risperidone; 9-OH-RIS, 9-hydroxyrisperidone; R/9-OH-RIS Ratio, risperidone/9-hydroxyrisperidone ratio; *p < 0.05

Of the 47 subjects who carried the CYP2D6*10 allele, 26 exhibited homozygous (TT) mutations, while the remaining 21 carried heterozygous mutations (CT). The plasma concentration of risperidone was significantly higher in subjects with homozygous mutations of CYP2D6*10 compared with heterozygous mutations, at each time point (Fig. 2; all *P* < 0.01). The difference in R/9-OH ratio was also significant at each time point (all *P* < 0.01), while 9-OH-RIS was only significantly different at week 2 (*Z =* -1.988, *P* = 0.046). The C/D ratio was significantly different between CYP2D6*10TT/CT carriers at weeks 4 and 8 (*Z =* -2.274, *P* = 0.025 and *Z =* -2.155, *P* = 0.031, respectively) but not at week 2. The change in the BPRS score from baseline to week 8 was significantly different between subjects with heterozygous or homozygous mutations (*Z = 4.667, P* = 0.040) (Table 4). We did not observe any significant differences in adverse effects between subjects with either mutation (all *P* > 0.05) (Table S2).

**Table 4:** Relationships between response to treatment and CYP2D6*10 allelic mutation

	*TT (26)*	*CT (21)*	*Statistics*	*P*
PANSS0 – week 4	20.5 (12.0, 28.2)	19.0 (10.5, 35.0)	1.357	0.645
PANSS0 – week 8	27.0 (19.0, 32.0)	30.0 (17.0, 39.5)	4.287	0.235
BPRS0 – week4	34.0 (28.8, 40.0)	32.5 (30.8, 34.2)	15.136	0.291
BPRS0 – week8	29.0 (27.0, 34.0)	28.0 (26.3, 30.0)	4.667	0.040
CGI-S0 – week4	4.0 (3.0, 4.0)	3.5 (3.0, 4.0)	− 1.392	0.164
CGI-S0 – week 8	3.0 (2.0, 3.0)	2.0 (2.0, 3.0)	−0.125	0.900
CGI-I2 – week 4	−0.5 (−1.0, 0.0)	0.0 (− 1.0, 0.0)	− 0.599	0.549
CGI-I2 – week 8	0.0 (−1.0, 1.0)	0.0 (− 1.0, 1.0)	−3.84	0.701

Data are presented as median (interquartile range)

PANSS0 - week 4 represents the change of PANSS score from 0 to 4 weeks

*Abbreviations*: *BPRS* Brief Psychiatric Rating Scale, *CGI* Clinical Global Impression, *CGI-S* Clinical Global Impression Severity, *CGI-I* Clinical Global Impression Improvement *PANSS* Positive and Negative Syndrome Scale

### Single nucleotide polymorphisms, plasma concentration of risperidone, clinical response, and adverse effects in relation to polymorphisms of C100T, C2851T, and G4181C

#### Demographic, clinical, and genetic characteristics

The demographics and disease-specific characteristics of subjects are stratified by single nucleotide polymorphism (SNP; i.e., *C100T*, *C2851T*, or *G4181C*) and presented in Table S3. The mean age was significantly higher in subjects with the *G4181C* polymorphism in the C/C genotype (*F =* -2.982, *P* = 0.003). No other significant differences were identified in age or gender between the polymorphisms (both *P* > 0.05). Additionally, there were no significant differences in plasma concentration of risperidone, PANSS, BPRS, or CGI at baseline between subjects with different polymorphisms (all *P* > 0.05).

#### Plasma concentration of risperidone in relation to single-nucleotide polymorphism

**Fig. 3:**
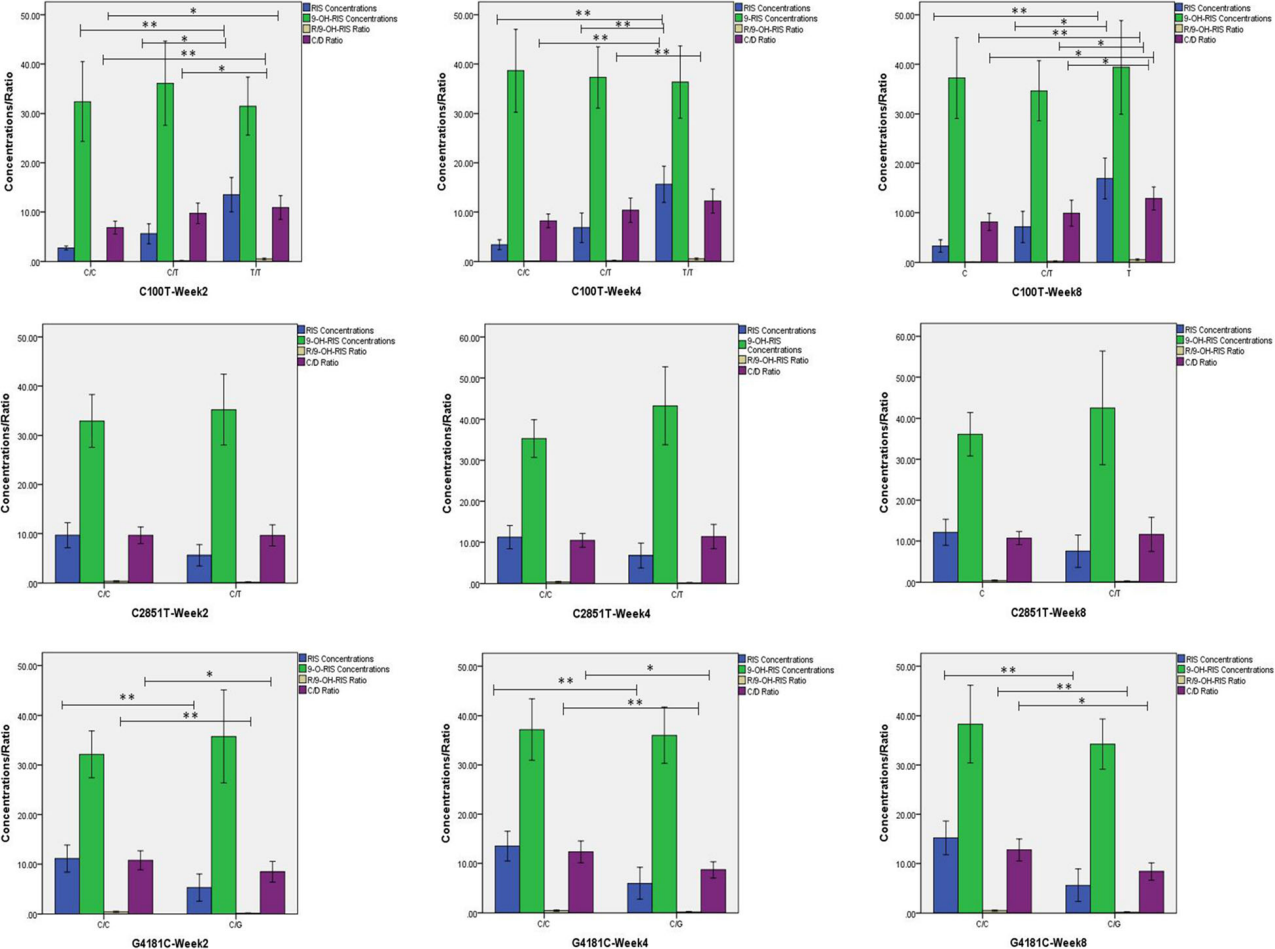
Plasma concentrations of risperidone in relation to polymorphisms of *C100T*, *C2851T*, and *G4181C.* Abbreviations: C/D, concentration/dose ratio; RIS, risperidone; 9-OH-RIS, 9-hydroxyrisperidone; R/9-OH-RIS Ratio, risperidone/9-hydroxyrisperidone ratio; *p < 0.05

Figure 3 illustrates the plasma concentration of risperidone with relation to the subjects respective SNP. Plasma concentrations of risperidone and the R/9-OH ratio were significantly higher in subjects with the T/T genotype compared with other genotypes in *C100T* at each time point (all *P* < 0.05). Further pairwise comparisons identified no other significant difference between the C/C and C/T genotypes in subjects with *C100T* (*P* > 0.05)*.* In terms of *G4181C*, a significantly higher plasma concentration of risperidone was identified in the C/C genotype compared with the G/C genotype at each time point (all *P* < 0.05). However, the 9-OH-RIS was not significantly different between genotypes in both *C100T* and *G4181C* at any time point (all *P* > 0.05). The C/D ratio was significantly different between subjects with *C100T* and *G4181C* polymorphisms (all *P* < 0.05) and between subjects with CC/TT and CT/TT genotypes in further pairwise comparisons at any time point (all *P* < 0.05). For the *C2851T* polymorphism, plasma drug concentration, R/9-OH, or C/D ratio were not significantly different for any genotype (all *P* > 0.05).

#### Clinical response and adverse effects in relation to single-nucleotide polymorphism

The associations between SNPs and clinical responses to risperidone treatment are presented in Table S4; there was no apparent association between improvement in PANSS, BPRS, or CGI scores at week 4 or 8 in relation to *C100T and C2851T* polymorphisms (all *P* > 0.05). However, for the G4181C polymorphism, improvement in CGI-S and CGI-I scores at week 4 were significantly different between the C/C and C/G genotypes (Z = 1.118, *P* = 0.016 and Z = -2.586, *P* = 0.010, respectively). Analysis of the predictive values of the alleles (CYP2D6*10, CYP2D6*2, or CYP2D6*65,) and SNPs (*C100T*, *C2851T*, and *G4181C*) with regard to clinical response (defined as 25% improvement in PANSS from baseline), identified 30 and 40 subjects as treatment responders at weeks 4 and 8, respectively. After controlling for confounding factors, regression analysis showed that plasma concentrations of risperidone at week 2 were a statistically significant predictor of clinical response (*B* = 0.642, *P* = 0.020 for response at week 4, *B* = 0.946, *P* = 0.003 for response at week 8). The relationship between adverse effects and SNPs are shown in Table 5. There were no significant differences in incidence of adverse metabolic effects or EPS between genotypes of the *C100T* polymorphism (all *P* > 0.05). In patients with the *C2851T* polymorphism, adverse metabolic effects, including weight change from baseline to week 4 (Z = -3.091, *P* = 0.008), changes in HDL from baseline to week 4 (Z = -2.073, *P* = 0.038) and week 8 (Z = 4.061, *P* = 0.035), and change in PRL from baseline to week 8 (Z = -2.179, *P* = 0.029), were significantly higher among patients with the C/T genotype compared with the C/C genotype. In contrast, the incidence of EPS, as assessed by ESRS or BAS, did not differ significantly in relation to genotype. With regards to the *G4181C* polymorphism, the change in GLU from baseline to week 4 (Z = -2.401 *P* = 0.016) and the TG level from baseline to week 4 (Z = 3.206, *P* = 0.001), were significantly higher in subjects with the C/C genotype compared with C/G, however, by week 8, this difference was not statistically significant (Z = -0.824, *P* = 0.410). There were no significant differences in any other adverse metabolic effects or the incidence of EPS, between genotypes of the *G4181C* polymorphism (all *P* > 0.05).

**Table 5:** Adverse effects among different single-nucleotide polymorphisms

	*C100T*					*C2851T*				*G4181C*			
	*C/C*	*C/T*	*T/T*	*Statistics*	*P*	*C/C*	*C/T*	*Statistics*	*P*	*C/C*	*C/G*	*Statistics*	*P*
Weight change from week 0 to 4	2.3(0.0, 4.3)	2.0(− 0.8, 4.0)	1.0(− 1.0, 2.5)	3.238	0.198	− 1.0(− 3.0, 1.0)	−4.0(−7.6, − 0.3)	−3.091	0.008	−1.3(− 3.0, 0.4)	−1.5(− 3.8, 1.0)	− 0.163	0.871
Weight change from week 0 to 8	− 3.0(− 4.4, − 0.5)	0.0(−5.0, 0.6)	− 1.0(−2.6, 1.2)	3.487	0.175	− 1.0(− 4.0, 1.0)	−1.5(− 4.0, 0.0)	− 0.533	0.594	− 1.0(− 4.0, 1.3)	0.0(−5.0, 0.0)	− 0.510	0.610
BMI change from week 0 to 8	− 1.1(− 1.8, − 0.2)	0.0(−1.7, 0.2)	− 0.4(− 1.0, 0.4)	4.010	0.135	− 0.4(− 1.6, 0.3)	(−1.6, 0.0)	− 0.680	0.496	− 0.4(− 1.6, 0.4)	0.0(− 1.7, 0.0)	− 0.469	0.639
GLU change from week 0 to 4	−0.1(− 0.4, 0.5)	−0.3(− 1.1, 0.2)	−0.5(− 1.3, 0.1)	2.776	0.250	− 0.3(− 0.9, 0.2)	− 0.4(− 1.2, − 0.1)	− 0.673	0.501	−0.6(− 1.2,-0.1)	− 0.1(− 0.5, 0.4)	−2.401	0.016
GLU change from week 0 to 8	−0.4(− 0.9, − 0.3)	−0.4(− 0.8, 0.6)	−0.2(− 0.7, 0.2)	0.106	0.899	− 0.2(− 0.8, 0.3)	−0.4(− 0.8, − 0.2)	0.447	0.847	−0.4(− 0.7, 0.1)	− 0.3(− 0.9, 0.6)	0.095	0.870
TG change from week 0 to 4	0.5(− 0.1, 1.7)	0.0(− 0.3, 0.5)	0.0(− 0.3, 0.4)	4.156	0.125	0.1(− 0.2, 0.6)	− 0.2(− 0.5, 0.5)	−1.497	0.134	−0.1(− 0.4, 0.4)	0.4(0.0, 0.9)	− 3.206	0.001
TG change from week 0 to 8	0.3(−0.2, 1.3)	− 0.1(− 0.4, 0.3)	0.0(− 0.2, 0.3)	2.879	0.237	0.1(− 0.3, 0.4)	0.0(− 0.5, 0.5)	−0.030	0.976	0.1(−0.5, 0.4)	0.1(− 0.2, 0.4)	−0.824	0.410
CHO change from week 0 to 4	−0.1(− 1.0, 1.1)	− 0.4(− 0.9, 0.3)	−0.3(− 0.8, 0.0)	2.813	0.067	− 0.2(− 0.8, 0.3)	−0.4(− 1.0, 0.0)	0.411	0.105	− 0.3(− 1.0, 0.1)	− 0.2(− 0.6, 0.4)	0.097	0.053
CHO change from week 0 to 8	−0.3(− 1.2, 0.5)	− 0.5(− 1.0, 0.3)	0.0(− 0.9, 0.4)	0.448	0.641	−0.4(− 0.9, 0.4)	−0.4(− 1.5, 0.5)	6.350	0.309	− 0.6(− 1.0, 0.4)	− 0.3(− 0.7, 0.3)	− 0.901	0.346
LDL change from week 0 to 4	0.2(−0.5, 0.8)	− 0.1(− 0.4, 0.3)	−0.1(− 0.5, 0.2)	2.430	0.095	0.0(− 0.5, 0.4)	0.0(− 0.3, 0.2)	0.316	0.413	− 0.2(− 0.5, 0.2)	0.0(− 0.4, 0.4)	0.009	0.069
LDL change from week 0 to 8	0.1(− 0.7, 0.8)	−0.4(− 0.6, 0.2)	−0.1(− 0.5, 0.5)	0.947	0.393	− 0.2(− 0.6, 0.4)	−0.1(− 0.7, 0.3)	0.997	0.708	− 0.2(− 0.7, 0.3)	0.0(− 0.6, 0.4)	− 1.243	0.214
HDL change from week 0 to 4	−0.1(− 0.2, 0.1)	−0.1(− 0.2, 0.0)	−0.1(− 0.3, 0.1)	0.227	0.839	− 0.1(− 0.2, 0.1)	−0.2(− 0.3, 0.0)	−2.073	0.038	−0.1(− 0.2, 0.0)	0.0(− 0.2, 0.0)	3.847	0.939
HDL change from week 0 to 8	−0.2(− 0.3, 0.0)	−0.1(− 0.3, 0.0)	−0.1(− 0.2, 0.1)	1.129	0.569	− 0.1(− 0.2, 0.1)	−0.2(− 0.4, 0.0)	4.061	0.035	0.0(− 0.2, 0.1)	−0.1(− 0.3, 0.0)	− 1.089	0.341
PRL change from week 0 to 4	−56.6(− 117.3, − 10.5)	−42.5(− 98.8, − 16.0)	− 28.6(− 108.8, − 0.7)	0.376	0.828	39.4(5.0, 82.6)	42.5(12.1, 131.4)	−0.786	0.432	50.3(5.9, 128.3)	39.5(12.3, 100.4)	4.276	0.584
PRL change from week 0 to 8	57.4(0.0, 131.5)	46.4(25.3, 78.1)	33.4(1.8, 66.2)	0.71	0.492	33.4(1.8, 64.3)	81.7(30.7, 133.7)	−2.179	0.029	37.2(18.0, 79.0)	40.8(7.1, 82.9)	−0.399	0.690
ESRS week 4	3	6	7	0.144	0.570	15	5	−0.094	0.578	12	3	−1.771	0.085
ESRS week 8	3	7	10	0.525	0.828	15	5	−0.094	0.578	13	4	−1.740	0.094
BAS week 4	1	4	3	0.741	0.693	4	3	−1.167	0.353	5	3	−0.152	0.598
BAS week 8	1	5	3	0.596	0.653	7	2	−0.124	0.634	4	5	−0.912	0.472

Data are presented as median (interquartile range). *Abbreviations*: *BAS* Barnes Akathisia Scale, *BMI* Body mass index, *CHO* Cholesterol, *ESRS* Extrapyramidal Symptom Rating Scale, *GLU* Glucose, *HDL* High-density lipoprotein, *LDL* Low-density lipoprotein, *PRL* Prolactin, *TG* Triglyceride

## Discussion

The present study demonstrates that among patients with schizophrenia, the *CYP2D6* genotype significantly influenced the plasma concentration of risperidone. This finding also suggests that among patients treated with risperidone, genotype may influence adverse drug reactions. However, the *CYP2D6* genotype exerts a slight effect on improvement of clinical symptoms.

The main finding of the current study is that the plasma drug concentrations of risperidone were significantly different among different *CYP2D6* alleles or SNPs. Similarly, significant differences were also observed between heterozygotes and homozygotes among the most common CYP2D6*10 alleles. Different *CYP2D6* alleles or SNPs were significantly associated with adverse drug effects, including adverse metabolic reactions and EPS. No obvious differences were observed between heterozygotes and homozygotes among the CYP2D6*10 allele. Only a slight difference was reported in the improvement of clinical symptoms among individuals with different *CYP2D6* genotypes.

Our study revealed that the *CYP2D6* genotype influenced the metabolism of risperidone, evidenced by increased plasma concentration and metabolic ratio (RIS/9-OH-RIS) [12, 16, 22]. Furthermore, our investigation of different alleles agree with the results of previous studies [12, 23], as well as a recent study involving children and adolescents with autism spectrum disorders which reported plasma concentrations of risperidone and RIS/9-OH-RIS to be significantly associated with CYP2D6*10, while plasma concentration of 9-OH-RIS was not found to be significantly correlated [13]. However, some studies have shown CYP2D6*10 to be associated with plasma concentrations of both risperidone and 9-OH-RIS, conflicting with our results [22, 23].

We found that after treatment, the plasma concentration of risperidone, was significantly different depending on the homo- or heterozygosity of CYP2D6*10 mutations, confirming previous studies that reported subjects who had homozygous mutations CYP2D6*10 to have higher plasma concentrations of risperidone compared with single-allele carriers [24, 25]. This may be attributed to differences in enzymatic activity; the enzymatic activity of the *CYP2D6* gene product is significantly lower in CYP2D6*10-homozygous subjects compared with wild-type homozygotes, while the CYP2D6*10-heterozygous subjects exhibit moderate enzyme activity [26].. Therefore, CYP2D6*10 is associated with lower enzymatic activity. Our study revealed that subjects with CYP2D6*2 have lower plasma concentrations of risperidone, indicating increased enzymatic activity after treatment, compared with the other two genotypes. Thus, there appears to be a significant relationship between *CYP2D6* genotype and *CYP2D6* activity; however, to the best of our knowledge, no study has directly compared *CYP2D6* activity between these three genotypes. Indeed, research regarding CYP2D6*65 and CYP2D6*2 is limited. Considering the small sample size of the present study, the differences that we have identified should be considered preliminary and interpreted with caution; further long-term large-scale research is warranted.

Significant associations were observed between SNPs (*C100T*, *C2851T*, and *G4181C*) and plasma concentrations of risperidone, RIS/9-OH-RIS ratio, and C/D ratio. To the best of our knowledge, this is the first exploratory research to investigate the relationships between *CYP2D6* SNPs, plasma concentration of antipsychotics, and clinical response relative to antipsychotic treatment, in patients with schizophrenia. Similar research involving psychiatric patients has shown the *CYP2D6* 1584C > G polymorphism as having a significant influence on thioridazine:mesoridazine plasma ratio, although the correlation between polymorphisms and clinical response was not investigated in this study [27]. We discovered that the *C100T* and *G4181C* polymorphisms were associated with differences in plasma concentration of risperidone and RIS/9-OH-RIS ratio. These findings indicate that even single-nucleotide mutations are sufficient to affect the activity of metabolic enzymes. Furthermore, the C/D ratio was obviously different between SNPs, suggesting that plasma clearance varies according to allelic variants. These factors may contribute to the clinical treatment response, and these findings provide new insight for individualized drug therapy.

We found CYP2D6*2 to be associated with increased improvement in clinical symptom, while CYP2D6*65 was found to be more associated with metabolic indicators. These observations contradict our hypothesis that carriers of CYP2D6*2 have higher enzymatic activity of *CYP2D6,* resulting in reduced plasma concentration of risperidone and therefore, reduced improvement of clinical symptoms. These findings are also inconsistent with previous research which has demonstrated lower enzymatic activity to be associated with an increased clinical response to risperidone treatment [14]. This contradiction may be attributed to the smaller sample size with the CYP2D6*2 allele. We did not detect a significant association between CYP2D6*10 and improvement of clinical symptoms, confirming the results of previous studies [28]. However, investigations regarding the relationships between genotype and clinical response are scarce and discrepant; studies involving psychiatric patients receiving risperidone have not identified any association between *CYP2D6* polymorphism and clinical improvement [15, 16]. Besides, it is difficult to compare studies including subjects with various combinations of genotypes [15, 16]. With regards to SNPs, we did not identify any significant differences in clinical response among the different single nucleotide variants, contrary to our hypothesis. The small sample size may have contributed to the lack of clarity, which is exacerbated by the lack of similar studies. Further studies with larger sample sizes are warranted to clarify the relationship between SNPs and clinical response.

Consistent with previous research, we found clear associations between genotype and adverse effects, including adverse metabolic reactions and EPS. A study involving children and adolescents receiving antipsychotics identified a significant difference in weight gain between patients with the CYP2D6*1/*4 genotype and who did not carry allele*4 [29]. Another study reported that patients with the CYP2D6 *1/*3 or *4 genotype, that were treated with atypical antipsychotics exhibited significantly larger percent change in body mass index (*p* < 0.0097) compared with those with a *1/*1 genotype [30]. In the present research, CYP2D6*10 and CYP2D6*2 were found to be associated with increased weight gain compared with CYP2D6*65, which contradicts some previous reports but agrees with others. A previous study suggested that individuals with the poor-metabolizer phenotype have increased plasma drug levels after treatment, resulting in serious antipsychotic-induced toxicity with consequent dose-dependent complications [31]. Another study confirmed that the poor-metabolizer phenotype was associated with an increased incidence of adverse effects of risperidone treatment [19]. Theoretically, CYP2D6*65 should be related to increased weight again while CYP2D6*2 should not be. Lane et al. reported a significant correlation between CYP2D6*10 and weight gain in patients receiving risperidone [32]. These findings are supported by the results of the present study, which also demonstrated a significant difference in the change of HDL from baseline in relation to genotype; CYP2D6*65 was associated with lower HDL levels following risperidone treatment compared with the other genotypes. The relationship between HDL and *CYP2D6* genotype has not been reported previously. In contrast, we found that in response to risperidone treatment, CYP2D6*65 was associated with higher levels of prolactin compared with the other alleles, particularly CYP2D6*10. Previous reports on the association between *CYP2D6* polymorphisms in relation to prolactin concentrations, are controversial. One study explored the impact of *CYP2D6* polymorphisms on the prevalence of risperidone-induced adverse effects, including hyperprolactinemia, revealed that the poor-metabolizer phenotype influenced the frequency of adverse effects and poor treatment compliance [19]. Although only assessed in a small cohort, another study revealed that *CYP2D6* ultra-rapid metabolism may contribute to increased prolactin levels in children, consist with our observations [33]. Also consistent with the results of a previous study [34], we found that the incidence of EPS was significantly higher among subjects with the CYP2D6*10 genotype. However, the predictive value of genotype for extrapyramidal side-effects is controversial [31, 35]. Some authors have suggested that “poor metabolizers” have significantly more pronounced or severe predisposing factors for the development of acute EPS [19, 34], which is supported by our finding that subjects carrying CYP2D6*10 or CYP2D6*65 are more likely to suffer EPS. With respect to SNPs, our study revealed that weight change and HDL or PRL alterations from baseline in response to treatment, differed significantly between subjects with *C2851T* wild-type compared with mutant, while GLU and TG differed significantly between subjects with *G4181C* homozygous mutations compared with heterozygous mutations. Previous studies on SNPs and their association with adverse reactions are scarce, with only one published report describing the association between the 1846G > A polymorphism of *CYP2D6* and extrapyramidal side effects [36]. Our results suggest that SNPs affect the clinical response, and occurrence of adverse reactions, to risperidone treatment. These data reveal a potential tool for predicting treatment efficacy and may also facilitate the prevention of adverse effects.

Collectively, the current study showed that polymorphisms of the *CYP2D6* gene had a significant effect on drug concentration and adverse drug effects. Previous studies have shown that polymorphisms of the *CYP2D6* gene display different enzyme activity including decreased, increased, or nonfunctional enzymatic activity [37]. Different enzymatic activity contributed to interindividual variability in plasma drug concentration, and subsequently, clinical outcomes [10]. Our study did reveal that poorly metabolizing genotypes, such as CYP2D6*10, were associated with more severe adverse effects, including weight gain and increased HDL and EPS, supporting the assumption that *CYP2D6* gene polymorphisms may be associated with different adverse reactions via alterations in enzyme activity. However, the slight improvement in clinical symptoms did not seem to be associated with *CYP2D6* genetic variations.

The present study has some limitations which should be acknowledged. Firstly, the sample size was small. Secondly, subjects were followed-up for only 8 weeks; therefore, we cannot comment on the effects of *CYP2D6* polymorphisms on the long-term clinical responses and side-effects, which may affect our conclusions. Additionally, patients who were non-compliant with treatment were excluded from the analysis and the study could be missing some key patient characteristics that influence drug treatment response. Finally, we only identified three significant single-nucleotide gene-effective mutation sites. Therefore, comparisons between more mutation sites could not be performed. Further verification through studies involving large sample sizes and comparing more genotypes are warranted.

## Conclusion

In summary, our study reveals the association between *CYP2D6* polymorphisms and the plasma concentration of risperidone, and that *CYP2D6* genotypes may serve as a predictor of adverse reactions, but not improvement of clinical symptoms, after risperidone treatment.

## Supplementary Information

**Additional file 1.**

## Data Availability

The datasets used are available from the corresponding author on reasonable request.

## Abbreviations

BMI: Body mass index
PANSS: The Positive and Negative Syndrome Scale
BPRS: Brief Psychiatric Rating Scale
CGI-S: Clinical Global Impression-Severity
CGI-I: Clinical Global Impression-Improvement
BAS: Barnes Akathisia Scale
CHO: Cholesterol
ESRS: Extrapyramidal Symptom Rating Scale
GLU: Glucose
HDL: High-density lipoprotein
LDL: Low-density lipoprotein
PRL: Prolactin
TG: Triglyceride
C/D: Concentration/dose ratio
RIS: Risperidone
9-OH-RIS: 9-hydroxyrisperidone
R/9-OH-RIS Ratio: Risperidone/9-hydroxyrisperidone ratio
