# Supplementary material for: Effect of CYP2D6 polymorphisms on plasma concentration and therapeutic effect of risperidone
Source: BMC Psychiatry. 2021 Feb 3;21:70. doi: 10.1186/s12888-020-03034-9 (PMC7856706; doi:10.1186/s12888-020-03034-9)
Supplement: Supplementary file 1 — Additional file 1. [file 12888_2020_3034_MOESM1_ESM.docx]

**Table S1.** **The details of individual PCR programs.**

| Primers |  | Exons | Size |
| --- | --- | --- | --- |
| Forward | GAGGGGTTGTGACTACATTA | 1,2,3,4 | 2671bp |
| Reverse | TCTCTGACGTGGATAGGAGG |  |  |
| Forward | AGGGGACATCTCAGACATGG | 5,6 | 941bp |
| Reverse | CCTTCTTGCCTCCTATGTTG |  |  |
| Forward | TAGGAGGCAAGAAGGAGTGT | 7 | 394bp |
| Reverse | GGGGACAGTCAGTGTGGTGG |  |  |
| Forward | TGTAAGCCTGACCTCCTCC | 8,9 | 1343bp |
| Reverse | AACATACCCCTGTCTCAAA |  |  |

**Table S2. Relationships between adverse effects and CYP2D6*10 allelic mutation.**

|  | *TT(26)* | *CT(36)* | *Statistics* | *P* |
| --- | --- | --- | --- | --- |
| Weight change from 0 to 4week | -1.0(-2.0,1.0) | -2.0(-3.0,1.0) | -0.747 | 0.455 |
| Weight change from 0 to 8week | -1.0(-3.0,2.0) | 0.0(-5.0,1.0) | -0.219 | 0.826 |
| BMI change from 0 to 8week | -0.4(-1.1,0.6) | 0.0(-1.7,0.3) | -0.187 | 0.622 |
| GLU change from 0 to 4Week | 4.6(4.1,5.0) | 4.6(4.4,5.3) | 0.734 | 0.453 |
| GLU change from 0 to 8Week | 0.0(-0.7,0.2) | -0.3(-0.9,0.6) | 1.518 | 0.962 |
| TG change from 0 to 4Week | 1.3(0.9,2.2) | 1.5(1.0,2.4) | -0.485 | 0.628 |
| TG change from 0 to 8Week | 0.1(-0.2,0.3) | -0.1(-0.3,0.2) | -1.082 | 0.279 |
| CHO change from 0 to 4Week | -0.2(-1.1,0.2) | -0.3(-0.6,0.2) | ·1.777 | 0.754 |
| CHO change from 0 to 8Week | -0.3(-0.9,0.5) | -0.3(-0.7,0.3) | 2.070 | 0.957 |
| LDL change from 0 to 4Week | -0.2(-0.5,0.2) | -0.1(-0.4,0.3) | 0.689 | 0.405 |
| LDL change from 0 to 8Week | -0.1(-0.5,0.7) | 0.0(-0.6,0.3) | 1.907 | 0.369 |
| HDL change from 0 to 4Week | -0.1(-0.2,0.1) | -0.1(-0.2,0.0) | -0.093 | 0.926 |
| HDL change from 0 to 8Week | 0.0(-0.2,0.1) | -0.1(-0.3,0.1) | 1.848 | 0.194 |
| PRL change from 0 to 8Week | 28.1(0.5,44.5) | 41.9(11.3,73.7) | 0.010 | 0.414 |
| ESRS 4Week | 4 | 2 | -1.281 | 0.227 |
| ESRS 8Week | 7 | 4 | -1.595 | 0.177 |
| BAS 4Week | 3 | 2 | -0.847 | 0.641 |
| BAS 8Week | 3 | 4 | -0.052 | 0.631 |

Data are presented as median (interquartile range).

Abbreviations: BAS, Barnes Akathisia Scale; BMI, body mass index; CHO, cholesterol; ESRS; Extrapyramidal Symptom Rating Scale; GLU, glucose; HDL, high-density lipoprotein; LDL, low-density lipoprotein; PRL, prolactin; TG, triglyceride.

**Table S3. Comparisons of demographic and disease-specific characteristics according to the C2851T, G4181C and C100T polymorphisms of CYP2D6***.*

|  |  | *C100T* | | | | |  | *C2851T* | | | |  | | *G4181C* | | | |
| --- | --- | --- | --- | --- | --- | --- | --- | --- | --- | --- | --- | --- | --- | --- | --- | --- | --- |
|  | *C/C(n=14)* | | *C/T(n=29)* | *T/T(n=33)* | *Statistics* | *P* | *C/C(n=55)* | | *C/T(n=20)* | *Statistics* | *P* | | *C/C(n=44)* | | *C/G(n=27)* | *Statistics* | *P* |
| A*g*e, years | 34.5(30.3,45.0) | | 44.0(34.0,55.5) | 50.0(36.5,54.0) | 5.375 | 0.068 | 45.0(33.0,52.0) | | 48.0(33.0,57.5) | 1.918 | 0.300 | | 50.5(38.5,55.8) | | 37.0(29.5,44.8) | -2.982 | 0.003 |
| Sex(M,F) | 5,9 | | 17,12 | 16,17 | 2.035 | 0.361 | 30,25 | | 8,12 | 1.241 | 0.324 | | 21,23 | | 16,12 | 0.607 | 0.476 |
| Dosage, mg/day | 5.0(4.0,6.0) | | 5.0(3.0,6.0) | 4.0(4.0,6.0) | 2.709 | 0.258 | 5.0(4.0,6.0) | | 5.0(4.0,5.8) | -0.620 | 0.535 | | 4.0(3.3,5.8) | | 5.0(4.0,6.0) | -1.850 | 0.064 |
| PANSS baseline | 89.0(82.3,95.0) | | 86.0(80.5,93.0) | 89.0(83.5,96.0) | 1.709 | 0.439 | 88.15±7.44 | | 89.50±9.23 | 0.793 | 0.516 | | 88.93±8.35 | | 86.93±7.26 | 0.124 | 0.300 |
| BPRS baseline | 45.5(41.8,49.3) | | 45.0(41.0,50.0) | 46.0(42.0,51.5) | 0.585 | 0.747 | 45.0(41.0,51.0) | | 46.0(42.0,51.5) | -0.492 | 0.622 | | 45.0(41.3,51.8) | | 45.0(41.0,50.0) | 0.736 | 0.314 |
| CGI-S baseline | 5.0(4.8,6.0) | | 5.0(5.0,5.0) | 5.0(5.0,6.0) | 2.002 | 0.368 | 5.0(5.0,6.0) | | 5.0(5.0,6.0) | -0.659 | 0.510 | | 5.0(5.0,6.0) | | 5.0(4.3,5.0) | 0.037 | 0.144 |
| CGI-Iweek2 | 3.0(3.0,3.0) | | 3.0(3.0,3.0) | 3.0(3.0,4.0) | 4.290 | 0.117 | 3.0(3.0,3.0) | | 3.0(3.0,4.0) | -0.751 | 0.452 | | 3.0(3.0,4.0) | | 3.0(3.0,3.0) | 8.650 | 0.143 |

Data are presented as median(interquartile range)

Abbreviations: BPRS, Brief Psychiatric Rating Scale; CGI, Clinical Global Impression; CGI-S, Clinical Global Impression Severity; CGI-I, Clinical Global Impression Improvement; PANSS, Positive and Negative Syndrome Scale.

**Table S4. Clinical response of risperidone treatment among different SNPS.**

|  |  |  | *C100T* |  |  |  | *C2851T* |  |  |  | *G4181C* |  |  |
| --- | --- | --- | --- | --- | --- | --- | --- | --- | --- | --- | --- | --- | --- |
|  | *C/C* | *C/T* | *T/T* | *Statistics* | *P* | *C/C* | *C/T* | *Statistics* | *P* | *C/C* | *C/G* | *Statistics* | P |
| PANSS0-Week 4 | 25.0(13.0,31.8) | 18.0(8.0,28.0) | 16.0(10.5,28.0) | 1.968 | 0.374 | 20.0(12.0,29.0) | 19.0(8.8,25.3) | 0.995 | 0.244 | 16.5(9.0,27.25) | 19.0(12.3,28.8) | -0.744 | 0.457 |
| PANSS0-Week 8 | 34.0(21.3,38.5) | 27.0(13.0,35.8) | 23.0(18.8,32.0) | 2.778 | 0.249 | 28.0(19.0,35.0) | 24.5(16.3,34.8) | 0.060 | 0.569 | 22.5(16.0,32.0) | 29.0(17.0,37.0) | -1.359 | 0.174 |
| BPRS0-Week4 | 13.0(9.8,16.5) | 12.0(7.5,18.0) | 12.0(9.0,13.5) | 0.567 | 0.753 | 12.0(8.0,15.0) | 13.0(8.5,13.8) | -0.060 | 0.952 | 12.0(8.0,14.0) | 12.5(8.0,16.0) | -0.411 | 0.171 |
| BPRS0-Week8 | 15.0(13.8,24.0) | 16.0(12.0,24.5) | 16.0(13.0,18.5) | 0.144 | 0.931 | 16.0(13.0,22.0) | 15.0(14.0,26.5) | -0.138 | 0.890 | 16.0(13.0,21.2) | 16.0(16.0,21.8) | -0.336 | 0.419 |
| CGI-S0-Week4 | 0.0(0.0,1.3) | 0.0(0.0,1.0) | 1.0(0.0,1.0) | 1.720 | 0.432 | 1.0(0.0,2.0) | 1.0(10,2.0) | -0.454 | 0.650 | 1.0(0.0,1.0) | 0.0(0.0,0.0) | -1.118 | 0.016 |
| CGI-S0-Week8 | 1.0(0.0,1.3) | 1.0(0.0,1.0) | 1.0(0.0,1.0) | 2.174 | 0.337 | 2.0(2.0,3.0) | 2.0(2.0,3.7) | -0.648 | 0.517 | 1.0(0.0,1.0) | 0.0(0.0,1.0) | -0.323 | 0.075 |
| CGI-I2 – week 4 | 0.0(0.0,0.3) | 0.0(0.0,1.0) | 1.0(0.0,1.0) | 4.892 | 0.087 | 0.0(0.0,1.0) | 0.0(0.0,1.0) | -0.041 | 0.967 | 0.0(0.0,1.0) | 0.0(0.0,0.0) | -2.586 | 0.010 |
| CGI-I2 – week 8 | 1.0(0.0,1.3) | 1.0(0.0,1.0) | 1.0(0.0,1.0) | 1.459 | 0.482 | 1.0(0.0,1.0) | 1.0(0.0,2.0) | -1.336 | 0.182 | 1.0(0.0,1.0) | 0.0(0.0,1.0) | -1.811 | 0.070 |

Data are presented as median (interquartile range).

PANSS0 - week 4 represents the change of PANSS score from 0 to 4 weeks

Abbreviations: BPRS, Brief Psychiatric Rating Scale; CGI, Clinical Global Impression; CGI-S, Clinical Global Impression Severity; PANSS, Positive and Negative Syndrome Scale.
